# Adrenergic-Angiogenic Crosstalk in Head and Neck Cancer: Mechanisms and Therapeutic Implications

**DOI:** 10.3389/froh.2021.689482

**Published:** 2021-06-08

**Authors:** Vui King Vincent-Chong, Mukund Seshadri

**Affiliations:** ^1^Center for Oral Oncology, Roswell Park Comprehensive Cancer Center, Buffalo, NY, United States; ^2^Department of Dentistry and Maxillofacial Prosthetics, Roswell Park Comprehensive Cancer Center, Buffalo, NY, United States

**Keywords:** HNSCC, adrenergic signaling, angiogenesis, drug repurposing, propranolol

## Abstract

Head and neck squamous cell carcinomas (HNSCC) are loco-regionally aggressive tumors that often lead to debilitating changes in appearance, speech, swallowing and respiratory function in patients. It is therefore critical to develop novel targeted treatment strategies that can effectively target multiple components within the tumor microenvironment. In this regard, there has been an increased recognition of the role of neural signaling networks as mediators of disease progression in HNSCC. Here, we summarize the current knowledge on the mechanisms of adrenergic signaling in HNSCC specifically focusing on neurovascular crosstalk and the potential of targeting the adrenergic-angiogenic axis through repurposing of FDA-approved drugs against HNSCC.

## Introduction

Head and neck squamous cell carcinomas (HNSCC) are loco-regionally aggressive tumors that result in debilitating functional and esthetic sequelae in approximately half a million individuals worldwide [1, 2]. Although chemoradiation and immunotherapy-based approaches have led to improved therapeutic benefit, treatment resistance remains a significant clinical challenge [2]. Additionally, a majority of these patients experience prolonged treatment-induced morbidities including severe xerostomia, dysphagia, loss of dentition, and mandibular osteoradionecrosis [3]. Clearly, there is a need to investigate novel therapies that can exhibit improved therapeutic efficacy against HNSCC with reduced toxicities and treatment-related complications in this patient population.

One strategy to accomplish this goal involves assessing the anticancer activity of existing Food and Drug Administration (FDA) approved drugs used for non-oncologic indications, a concept termed as drug repurposing or repositioning [4, 5]. Given the significant costs associated with drug development, an informed approach focused on identifying and evaluating existing FDA-approved agents that target critical pathways implicated in development, progression or treatment resistance in HNSCC would be beneficial. In this context, there has been an increased recognition of the role of neural signaling networks as mediators of disease progression and therapeutic resistance in several solid tumors including HNSCC [6–8]. In this article, we summarize the current knowledge on the neurovascular talk in HNSCC specifically focusing on the adrenergic signaling within the head and neck tumor microenvironment. The rationale for targeting the adrenergic-angiogenic axis through repurposing of FDA-approved neuroscience drugs against HNSCC is presented.

## Neural Regulation of Cancer

The role of nerves in cancer patients, particularly in the context of cancer pain, has been long recognized [9, 10]. Sympathectomy (localized surgical interruption or removal of nerve fibers/ganglions) has been used to alleviate pain in cancer patients since the 1940s [9]. A study by Batkin et al. in 1970 showed that denervation of sciatic nerves resulted in a reduction in take of neuroblastomas in mice [10]. The infiltration of tumors by growing nerves termed as neoneurogenesis or axonogenesis has been linked to tumor progression [6, 7]. Recent landmark publications in prostate, pancreas and gastric cancers have demonstrated that nerves are not just passive players in carcinogenesis or tumor progression but an integral part of the tumor microenvironment [11–13]. Sympathectomy has been shown to prevent growth and metastasis of transplanted tumors and transgenic models of prostate cancer [11]. Surgical or pharmacologic denervation has been shown to reduce tumor incidence and progression in gastric cancer and enhance chemotherapeutic efficacy [12]. However, the role of nerves in tumor initiation or progression in HNSCC has not been systematically examined until recently.

## Neural Signaling and Adrenergic Stress in HNSCC

Head and neck cancers were among the first set of cancers that showed propensity to grow along nerves [14, 15]. Perineural invasion (PNI) is a distinct route of tumor spread that is recognized as a key pathologic feature of many cancers including HNSCC [16]. Over the last decade, experimental studies have implicated the autonomic nervous system, specifically, adrenergic signaling axis in oral cancer progression [17–21]. Chronic stress induced through physical restraint has been shown to promote cancer progression in a mouse model of HNSCC through increased norepinephrine (NE) which upregulates vascular endothelial growth factor (VEGF) and matrix metalloproteinase (MMP2) levels [17, 18]. Adrenergic stimulation has been shown to upregulate interleukin-6 (IL-6) in HNSCC via β-adrenergic receptor (ADRB) activation [19]. Activation of ADRB signaling has also been shown to promote tumor progression and epithelial-to-mesenchymal transition (EMT) in HNSCC [20]. Using the 4NQO carcinogen-induced model of oral squamous cell carcinoma (OSCC), Valente et al. have shown that baseline levels (prior to 4NQO exposure) of NE, cortisone and neurotrophins such as brain-derived neurotrophic factor (BDNF) in normal tongue can be predictive of cancer occurrence in rats exposed to 4NQO [22]. Amit et al. have recently examined the significance and mechanisms involved in neuron reprogramming in head and neck cancer [8]. Using *Krt5*^Cre^*Trp53*^*flox*/*flox*^ mice, the authors demonstrated increased nerve density in p53 deficient tumors compared to wild type p53 controls implicating the loss of p53 in epithelial cells with neuritogenesis during oral carcinogenesis. The authors showed that loss of *TP53* leads to phenotypic trans-differentiation of sensory nerves to adrenergic nerves and regulates cancer associated neurons via extracellular vesicle derived signals [8].

In the clinical setting, HNSCC patients have increased circulating levels of NE associated with their bio-behavioral symptoms and anxiety levels [23]. Multiple studies have shown that β-adrenergic receptor-2 (ADRB2) is highly expressed in HNSCC compared to normal epithelium and has been associated with alcohol and tobacco use [24, 25]. The prognostic implications of ADRB2 expression, however, are unclear. Shang and colleagues examined ADRB2 expression in 65 human OSCC specimens and 10 normal oral mucosa samples and observed a higher expression of ADRB2 in OSCC that was positively correlated with tumor size, clinical stage and lymph node metastasis [24]. Similarly, increased TH+ nerve density was associated with lower recurrence-free survival in OSCC [8]. In contrast, strong ADRB2 expression in Brazilian OSCC patients was associated with improved overall survival and cancer-specific survival compared to patients with weak/negative ADRB2 expression [25]. Perhaps due to the global epidemiology of OSCC (higher prevalence in Asia and South East Asia compared to North America or Europe), the prognostic significance of ADRB2 expression in North American or European HNSCC patients has not been reported. However, Amit et al. have reported on the role of adrenergic signaling in 70 head and neck cancer patients treated at MD Anderson Cancer Center (Texas, United States). Although ADRB2 staining was not performed, the authors showed that increased TH+ nerve density was associated with lower overall and recurrence free survival in their patient population [8].

## Adrenergic-Angiogenic Crosstalk in the Tumor Microenvironment

Angiogenesis is one of the hallmarks of cancer and initiation of the angiogenic switch is recognized as an early and critical event in head and neck cancer [26, 27]. Several studies have shown that overexpression of vascular endothelial growth factor (VEGF) has been associated with poor prognosis in HNSCC [28–31]. Although a considerable body of literature exists on the mechanism(s) of interactions between tumor cells and blood vessels, the literature on reciprocal neurovascular interactions in tumors, especially in HNSCC is limited.

**Figure 1:**
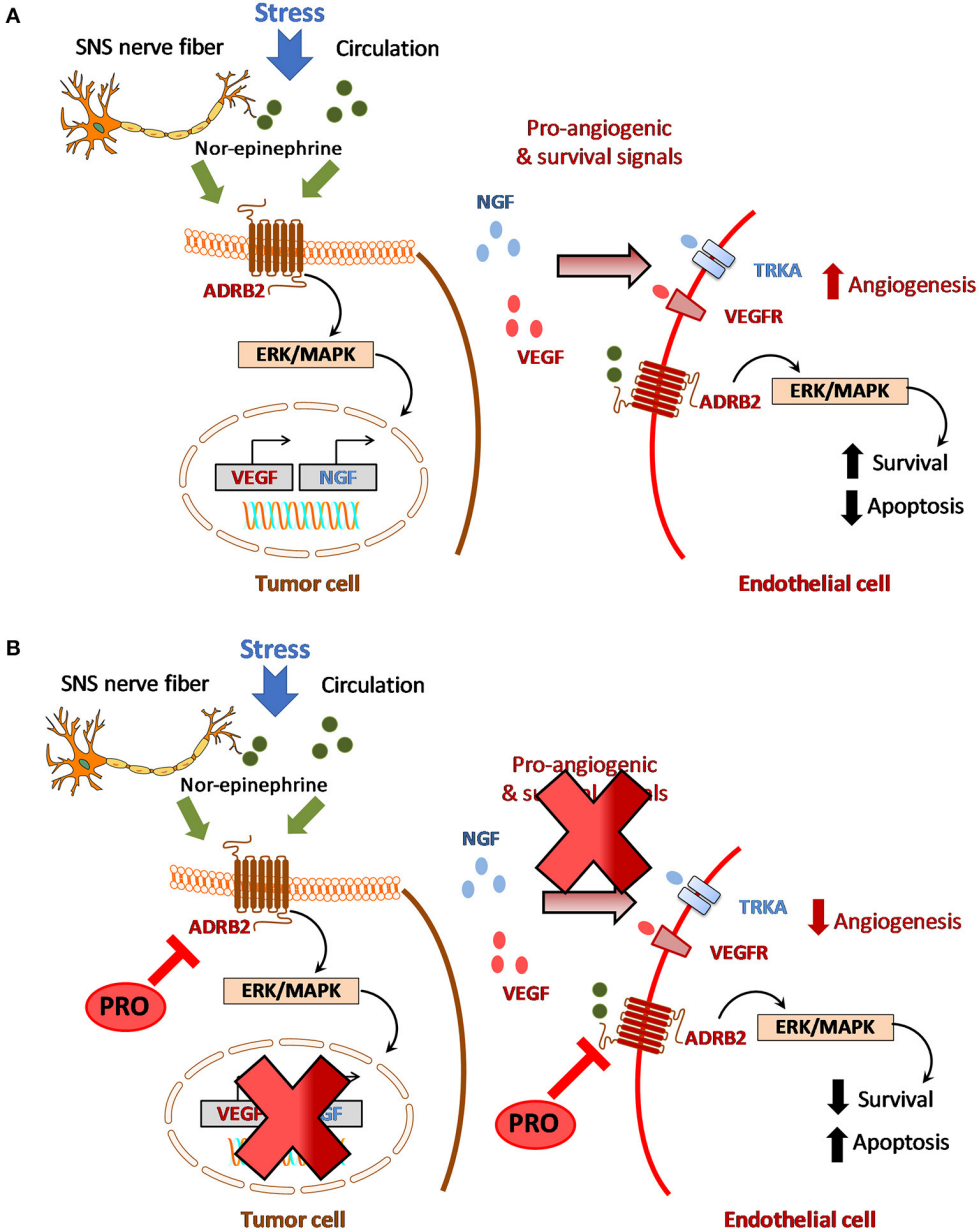
Crosstalk between adrenergic signaling and angiogenesis in HNSCC. **(A)** Stress induced activation of beta-adrenergic signaling within the tumor microenvironment which in turn promotes angiogenesis and disease progression. **(B)** Hypothetical model for targeting adrenergic-angiogenic axis in HNSCC using propranolol.

The cross talk between tumor cells, endothelial cells and nerves is mediated by growth factors such as nerve growth factor (NGF) which can regulate VEGF and matrix metalloproteinases (MMPs) within the microenvironment [32–35]. NGF is one of the well-characterized neurotrophins that can serve as autocrine factor to tumor cells [36, 37]. NGF binds to its low affinity receptor, NGFR (p75NTR) or the high affinity receptor, Tropomyosin-related kinase (TrkA) [38, 39]. Work by Ye et al. and Kolokythas et al. has shown that NGF is a critical factor that contributes to oral carcinogenesis [40, 41]. Expression of NGF can induce neovascularization around the nerves in turn promoting tumor growth and proliferation [42]. Conversely, VEGF can induce endothelial cells to secrete collagenase contributing to degradation of the basement membrane, a critical step in PNI, vascular invasion and metastatic dissemination [16, 42]. Common to these interactions is adrenergic signaling as ADRB2 is expressed both on tumor cells and endothelial cells and activation of adrenergic signaling promotes tumor cell survival, drive angiogenesis through several downstream signaling pathways (Figure 1A). ADBR2-mediated signaling (through local release of NE from SNS nerve fibers or from circulation) in tumor cells can upregulate NGF production which in turn can stimulate NGFR/TrkA signaling in an autocrine loop to promote cell survival by preventing apoptosis [13]. Chronic stress-induced release of neurotransmitters can activate ADRB2 and upregulate VEGF levels resulting in enhanced tumor vascularization and aggressive tumor growth [17, 43]. Activation of β-adrenergic signaling through NE has been shown to promote epithelial to mesenchymal transition (EMT) through upregulation of MMP2/9 and VEGF thereby enhancing the invasive and metastatic properties of tumor cells [17, 20]. β-adrenergic signaling is also involved in the regulation of hypoxia [44, 45]. It has been shown that β-adrenergic receptors are fundamental regulators of hypoxia and necessary for hypoxia-inducible factor 1 alpha (HIF-1α) accumulation [44]. In pancreatic cancer cells, binding of NE to ADRB2 has been shown to upregulate HIF-1α expression through Akt and ERK pathways [45]. While the interplay between tumor angiogenesis and hypoxia is well-known, the role of ADBR2 in and regulating tumor hypoxia especially through HIF-1α signaling has not been extensively studied in head and neck cancer and warrants further research. Nevertheless, these observations highlight the cross talk between the vascular and neural components within the HNSCC tumor microenvironment.

## Targeting the Adrenergic-Angiogenic Axis in HNSCC

### Repurposing Neuroscience Drugs—A Case for Propranolol

Given the role of adrenergic signaling in HNSCC, it would be reasonable to postulate that directly targeting adrenergic signaling in tumor cells or indirectly targeting neuro-vascular interactions (e.g., ADRB2-NGF-VEGF signaling) within the tumor microenvironment could have significant therapeutic benefit in HNSCC. However, safety concerns with anti-NGF antibodies [46] have hampered their clinical use in HNSCC patients. Given the huge cost and time-constraints associated with pharmaceutical development of novel agents, drug repurposing is an attractive approach that can enable successful identification and development of agents that can target the adrenergic-angiogenic axis in head and neck cancer. The availability of pharmacologic and toxicology data of these FDA-approved agents in humans can accelerate clinical evaluation of promising agents [4, 47]. In this regard, the non-selective beta-blocker, propranolol (PRO) is currently being investigated for its therapeutic potential against cancer [48]. PRO is FDA-approved for treating patients with variety of conditions ranging from hypertension and cardiac failure to neurological disorders including anxiety, migraines, tremors and glaucoma [49, 50]. In this section, we summarize the current preclinical and clinical evidence on the effects of PRO on adrenergic signaling and angiogenesis in HNSCC.

PRO has been shown to inhibit NE stimulated migration and invasion of HNSCC *in vitro* [20]. In nasopharyngeal carcinoma (NPC) cell lines, PRO has been shown to inhibit NE-induced MMP-2/9 and VEGF [17]. In Epstein-Barr virus (EBV) associated nasopharyngeal carcinoma, latent membrane protein 1 (LMP1) is the viral oncogene that promotes invasion and metastasis through effects on MMP-2/9 [51, 52]. Using EBV positive (clone 13) and EBV negative (clone 39) of LMP1 expressing HONE-1 cells, Yang et al. showed that NE stimulated the release of VEGF, MMP-2, and MMP-9 in both NPC cells independent of their EBV status. While the direct effects of EBV oncoproteins including LMP1 on adrenergic signaling is unclear, the study showed that both clones of the NPC cell line expressed ADRB2 and were inhibited by PRO through downregulation of MMP-2/9 expression [17]. Similarly, the forkhead box (FOXA) family of transcription factors have been implicated in the biology of NPC [53, 54]. Overexpression of FOXA1 has been shown to suppress proliferation, migration, and invasion of NPC cells in culture [53]. In NPC patients, FOXA1 expression has been correlated with prolonged disease-free survival and overall survival [54]. However, the effects of PRO on FOXA1 signaling has not been previously reported.

Preclinical studies have also examined the interaction between PRO and standard of care chemo- and radiation therapy in HNSCC. Wolter et al. have shown that PRO reduces HNSCC viability, inhibits VEGF production and can enhance the efficacy of cisplatin and radiation against HNSCC cells [55]. Recently, Lucido et al. have shown that PRO exhibits potent antitumor activity against human papillomavirus positive (HPV+) HNSCC that is mediated by a reduction of mitochondrial oxidative phosphorylation [56]. In the study, PRO in combination with chemoradiation resulted in inhibition of primary tumor growth and reduction in metastases.

And finally, studies have also demonstrated the antiangiogenic effects of PRO in experimental tumor models. PRO has been shown to suppress angiogenesis by inhibiting proliferation, migration and differentiation of endothelial cells *in vitro* [57]. Blockade of ADBR2 signaling by PRO inhibits VEGF induced phosphorylation of VEGFR2, extracellular signal-regulated kinase-1/2 (ERK) and pro-MMP2 secretion. PRO exhibits antiangiogenic effects at non-toxic concentrations (<50 μM) and potentiates the antiangiogenic and therapeutic efficacy of chemotherapeutic agents, 5FU and taxol [58]. PRO has been shown to inhibit growth, decrease vessel density and lower VEGF, MMP2/9 levels in neuroblastomas [59] and repress tumor growth in hemangiomas through hypoxia-inducible factor-1 alpha (HIF-1α) and STAT3 signaling [60]. Collectively, these preclinical observations highlight the therapeutic potential of PRO in targeting adrenergic-angiogenic signaling in HNSCC (Figure 1B).

Repurposing PRO for use in HNSCC patients presents an attractive strategy considering the cost of the drug and wealth of pharmacologic and toxicologic data that exists in humans. However, the existing clinical evidence from epidemiologic or retrospective analyses regarding PRO use in HNSCC is conflicting. Chang et al. have shown that long-term PRO use (>1,000 days) was associated with a reduction in risk of HNSCC (HR: 0.58; CI: 0.35–0.95) [61]. In contrast, an observational study in 1,274 patients conduced in South Korea showed that post diagnosis beta-blocker use was associated with decreased survival and increased recurrence in HNSCC patients [62]. A recent meta-analysis of epidemiologic and perioperative studies suggests that benefits associated with beta-blockers are likely to vary across patients with different tumor sites [63].

## Conclusions

In summary, neuronal programming and neurovascular interactions represent relatively understudied mechanisms that contribute to malignant progression in HNSCC. The literature presented in this review highlight the importance and therapeutic potential of targeting adrenergic signaling pathways within the head and neck tumor microenvironment. However, additional investigation to better understand the role of adrenergic-angiogenic cross talk in head and neck cancer and the potential of targeting this axis using PRO in the current treatment paradigm for HNSCC is warranted. In this regard, 3D organoid models and organoid co-culture systems can serve as a useful platform to dissect the mechanisms of interaction between tumor cells, neurons and endothelial cells and to screen therapeutic agents that can effectively target the adrenergic-angiogenic axis in head and neck cancer. Although limited, the published preclinical evidence on the activity of PRO against HNSCC is encouraging. Studies should therefore investigate the activity of PRO in combination with chemoradiation and immune checkpoint blockade using clinically relevant orthotopic models of HNSCC. Given the known effects of PRO on tumor cells, neural signaling, and blood vessels, such studies could employ clinically relevant imaging methods (e.g., MRI, PET) to assess the metabolic, vascular and hypoxic profiles of tumors. Integration of imaging phenotypes with genomic data and response to PRO would enable identification of patients that could benefit from the addition of PRO to existing standard of care regimens. Investigation into the potential chemopreventive effects of PRO in carcinogen-induced models of HNSCC could also be insightful. Such studies could serve to accelerate the clinical translation of a relatively inexpensive and a readily available drug to treat these esthetically and functionally debilitating cancers.

## Author Contributions

MS designed and drafted the manuscript. VKV-C and MS edited the manuscript, critically revised the content, and approved the final submitted version of the manuscript. All authors contributed to the article and approved the submitted version.

## Conflict of Interest

The authors declare that the research was conducted in the absence of any commercial or financial relationships that could be construed as a potential conflict of interest.
